# Insights Into the Albinism Mechanism for Two Distinct Color Morphs of Northern Snakehead, *Channa argus* Through Histological and Transcriptome Analyses

**DOI:** 10.3389/fgene.2020.00830

**Published:** 2020-09-18

**Authors:** Aiguo Zhou, Shaolin Xie, Yongyong Feng, Di Sun, Shulin Liu, Zhuolin Sun, Mingzhi Li, Chaonan Zhang, Jixing Zou

**Affiliations:** ^1^Joint Laboratory of Guangdong Province and Hong Kong Region on Marine Bioresource Conservation and Exploitation, College of Marine Sciences, South China Agricultural University, Guangzhou, China; ^2^Guangdong Laboratory for Lingnan Modern Agriculture, South China Agricultural University, Guangzhou, China; ^3^Independent Researcher, Guangzhou, China

**Keywords:** *Channa argus*, albino, histology, transcription characterization, gene expression

## Abstract

The great northern snakehead (*Channa argus*) is one of the most important economic and conservational fish in China. In this study, the melanocytes in the skin of two distinct color morphs *C. argus* were investigated and compared through employment of the microscopic analysis, hematoxylin and eosin (H&E) and Masson Fontana staining. Our results demonstrated the uneven distribution of melanocytes with extremely low density and most of them were in the state of aging or death. Meanwhile, there was no obvious pigment layer and melanocytes distribution pattern found in the albino-type (AT), while the melanocytes were evenly distributed with abundance in the bicolor-type (BT). The transcriptome analysis through Illumina HiSeq sequencing showed that a total of 34.93 Gb Clean Data was obtained, and Q30 base percentage reached 92.66%. The BT and AT northern snakeheads transcriptome data included a total of 56,039,701 and 60,410,063 clean reads (*n* = 3), respectively. In gene expression analyses, the sample correlation coefficients (*r*) were ranged between 0.92 and 1.00; the contribution of PC1 and PC2 were 50.25 and 13.73% by using PCA cluster analysis, the total number of DEGs were 1024 (559 up-regulated and 465 down-regulated), and the number of annotated DEGs was 767 (COG 172, KEGG 262, GO 288, SwissProt 548, Pfam 579 and NR 765). Additionally, 46,363 ± 873 and 44,947 ± 392 single nucleotide polymorphisms (SNPs) were compiled via genetic structure analysis, respectively. Ten key pigment-related genes were screened using qRT-PCR. And all of them revealed extremely higher expression levels in the skin of BT than those of AT. This is the first study to analyze the mechanism of albino characteristics of *Channa* via histology and transcriptomics, and also provide the oretical and practical support for the protection and development of germplasm resources for *C. argus*.

## Introduction

Being the largest number of existing vertebrate subphylums, the fish have over 32,000 species that are distributed across all of the aquatic resources worldwide (Nelson et al., 2016). Meanwhile, a lot of colorful fish varieties have been formed over a long period of natural selection and geographic evolution, some of them are for courtship and reproduction, some are for defense, some are for predation, some are for adapting to the environment, etc. (Keenleyside, 2012; Johnsson, 2019). The formation of final body color model is based on the movement and interaction among the pigment cells (Irion et al., 2016; Bian et al., 2019). Previously, the body colors of zebrafish have been thoroughly studied, which showed that the dark band area is mainly dominated by melanocytes and iridescent cells, while the body color is formed by the interaction of melanocytes, iridescent and yellow pigment cells in adult zebrafish (Frohnhöfer et al., 2013; Patterson and Parichy, 2013; Mahalwar et al., 2014; Fadeev et al., 2016).

As an important and one of the most common phenomenon of fish genetic variation, albinism is often stably inherited and finally unique varieties are formed. For instance, the albino of *Salvelinus fontinalis* can be stably inherited (Pettis, 1904). Further, the formation of zebrafish body color model also has a certain genetic mechanism, including cell–cell interaction band and agouti signaling protein (ASIP)-mediated formation mechanism (Ceinos et al., 2015). The albino-type (AT) *Channa argus* also showed a stable inheritance with the white body color over the generations. At the same time, the AT *C. argus* is only found in the Jialing River, China (105.05E, 29.58N) (Zhou et al., 2018, 2019). Our previous study showed that the AT has higher protein content and lower fat content in body composition than that of the bicolor-type (BT; Wang et al., 2018), which implies a higher nutritional value of the AT. Meanwhile, the potential ornamental values of BT are increasing day by day.

A previous study has classified the AT as one of a subspecies of *C. argus* (Shih, 1936), but our recent studies evidenced through molecular markers that the AT should be served as an albino of the BT *C. argus* (Zhou et al., 2017a,b, 2018, 2019), which might assist to modify the previous classification of this species. In order to further clarify the albino characteristic mechanism of two distinct color morphs *C. argus*, we first observed the distribution and differences of skin melanocytes by H&E staining and melanin staining in this study. Then, the key differentially expressed genes (DEGs) related to albinism have been screened by using high-throughput sequencing, their genetic expressions in the skin have been further validated by qRT-PCR. In a nutshell, this study aims to provide certain theoretical and practical basis for the protection and development of *C. argus* diversity in fresh-water resources.

## Materials and Methods

### Sample Collection

Two distinct color morphs of *C. argus* were collected from the Jialing River, in Neijiang city (105.05E, 29.58N), Sichuan province, China. The lengths of fish were 15 ± 1.3 cm and 12 ± 0.8 cm.

### Ethics

The animal study was reviewed and approved by the Animal Care Committee of South China Agricultural University (Guangzhou, China).

### Sample Treatments for Histological Observation

The fresh fish were anesthetized with a lethal dose of MS-222 anesthetic (300 ppm). Then the scales on the surface of the fish were gently scraped off using a scalpel, and the skins were rinsed and cleaned. The body skins were gently cut into small pieces of 5–10 mm using a scalpel and tweezers, and washed with phosphate buffer. The temporary slides were prepared by using a dissecting needle. Meanwhile, the small sections of skin were fixed in 4% paraformaldehyde for more than 24 h, and then placed in a dehydration box. The box was put into the dehydrator for gradient dehydration with parameters set at 75% alcohol for 4 h, 85% alcohol for 2 h, 90% alcohol for 2 h, 95% alcohol for 1 h, anhydrous ethanol I for 30 min, anhydrous ethanol II for 30min, alcohol benzene for 5-10 min, xylene for I5-10 min, Xylene II for 5-10 min, Wax I for 1 h, Wax II for 1 h, and Wax III for 1 h. Then waxes were embedded in the embedding machine to obtain the 4 mm baking sheets, for H&E staining melanin staining. The dyeing steps include the dewaxing of paraffin sections (the slices were placed turn by turn in xylene I for 20 min, xylene II for 20 min, absolute ethanol I for 10 min, absolute ethanol II for 10 min, 95% alcohol for 5 min, 90% alcohol for 5 min, 80% alcohol for 5 min, 70% alcohol for 5 min and washed with distilled water). For H&E staining, the nuclear dyeing of slices was performed for 3–8 min with the harris hematoxylin solution, washed with water and differentiated a few seconds using 1% hydrochloric acid alcohol, washed with water and turned to blue with 0.6% ammonia, and washed with water, then the cytoplasmic dyeing of slices was performed with eosin solution for 1–3 min. And for Masson-Fontana, the slices were performed with silver ammonia solution and ark treatment for 12–18 h, washed with water, then dealt with 5% sodium thiosulfate treatment for 2 min, and counterstained with Van Giesan dye solution for 20 s, washed with water. Both the dyed slices were immersed in 95% alcohol I for 5 min, 95% alcohol II for 5 min, absolute ethanol I for 5 min, absolute ethanol II for 5 min, xylene I for 5 min, and xylene II for 5 min. The dehydrated slices were then taken out, dried, and sealed with neutral gum. Finally, the slices were observed and photographed with a Leica microscope (DM2500, Wetzlar, Germany), and photographed with a CCD shooting system and measured with the system’s own software.

### Sample Preparation and Illumine Sequencing

As per standard instructions from the Ministry of Environment, China, a total of 18 individuals (AT and BT) from each species were used for experimental purposes. Total RNA was extracted from the spin tissues of two distinct color morphs of *C. argus* using Trizol Reagent (Invitrogen, Carlsbad, CA, United States) according to the manufacturer’s instructions. The processed RNA was checked for purity and integrity using Nanodrop-2000 spectrophotometer (Thermo Fisher Scientific, Wilmington, DE, United States), Qubit 2.0 (Thermo Fisher Scientific, United States) and the Bioanalyzer 2100 (Agilent Technologies, Santa Clara, CA, United States). Each species was investigated with three pools, and each pool included three individual samples. The mRNA-seq library was constructed using the mRNA-seq sample preparation kit (Illumina, San Diego, CA, United States). In the process, the total RNA was treated with DNase I and magnetic beads with Oligo (dT) to obtain and purify poly (A+) mRNA. The purified mRNA was fragmented using the DNA fragmentation kit (Ambion, Austin, TX, United States) prior to cDNA synthesis. The short fragments of mRNA were used to transcribe first-strand cDNA using reverse-transcriptase (Invitrogen) and random hexamer-primers. The synthesis of second-strand cDNA was accomplished using DNA polymerase I (New England BioLabs, Ipswich, MA, United States) and RNase H (Invitrogen). Then the double-stranded cDNA was purified using AMPure XP beads. Subsequently, the purified double-stranded cDNA was end-repaired using T4 DNA polymerase, the Klenow fragment, and the T4 polynucleotide kinase (New England BioLabs). The end-repaired cDNA fragments were connected with PE (Paired-end) Adapter Oligo Mix using T4 DNA ligase (New England BioLabs) at room temperature for 15 min. The selection of fragment size was analyzed also using AMPure XP beads. Finally, the cDNA library was obtained by using PCR enrichment. The cDNA library was preliminarily quantified using Qubit 2.0, and diluted carefully, then the insert fragment size detected using Bioanalyzer 2100, and the effective concentration of the cDNA library accurately quantified using qRT-PCR to ensure library quality. Finally, different cDNA libraries were pooled into flow cells, and were sequenced using Illumina high-throughput sequencing platforms (HiSeq/MiSeq) after cBOTs were clustered.

### Analysis of Sequencing Data

The raw data was filtered, and the linker sequence and low-quality reads were removed to obtain the clean data. Then the sequence of clean data was aligned with the reference genome (GeneBank: SRP078899) by using HISAT2 to obtain the mapped data. The library quality was controlled, and insert size and randomness were tested to obtain the comparison efficiency and coverage area of each sample. In order to splice the complete and accurate genes, the transcript was remodeled using the StringTie algorithm. In analyzing the gene structure, the potential SNP locis in gene regions was identified and found using SAM tools software (Li et al., 2009). The alternative splicing was analyzed using Astalavista software^1^. The gene expression was analyzed by using StringTie software, and FPKM (Fragments Per Kilobase of transcript per Million fragments mapped) was act as an indicator for measuring the gene expression (Trapnell et al., 2010):

FPKM=cDNA⁢fragments/mapped⁢reads⁢(millions)×transcript⁢length⁢(kb)

cDNA Fragments, the number of fragments aligned on a transcript; Mapped fragments (Millions), the total number of fragments aligned on the transcript (10^6^); transcript length, the transcript length (10^3^bp).

The correlation between samples was evaluated using Pearson’s Correlation (Schulze et al., 2012). The DESeq2 software was used for screening DEGs, the selection criteria were log2 |Fold Change| ≥ 1 and False Discovery Rate (FDR) < 0.05. The overall distribution of expression levels and fold change were analyzed by using MA and Volcano map. The cluster analysis heatmap of DEGs was constructed. Swiss-Prot, Gene Ontology (GO), Kyoto Encyclopedia of Genes and Genomes (KEGG), Cluster of Orthologous Groups (COG), EuKaryotic Orthologous Groups (KOG), Pfam, and Non-Redundant (NR) analysis were used for gene feature annotation, classification and enrichment.

### qRT-PCR Analysis

The qRT-PCR primers were designed (Table 1), and their specificities were examined by conventional PCR and melting curve analyses. β*-actin* gene was used as the internal control (Zhou et al., 2018). A three-step method was used for the following amplification scheme on an iQ5 Real-time PCR instrument (Bio-Rad, United States): incubation for 2 min at 95°C, followed by 45 cycles of 10 s at 95°C, 34 s at 60°C for optimized temperatures for specific genes, and 30 s at 72°C. The melting curve temperature ranged from 60 to 98°C, and analysis was performed to confirm the presence of a single applicant. Relative expression was determined using the 2^(–Δ^ ^Δ^ ^Ct)^ method (Livak and Schmittgen, 2001).

**TABLE 1:** Eight target genes and β*-actin* validation using Real-time PCR analysis.

**Gene primer**	**Primer sequence (5′–3′)**	**TM (°C)**	**Amplification efficiency (%)**
*TYR*-F	CTGACTAACTGGGAGAATGAGATAAG	60.8	99
*TYR*-R	CCACTTTCCATGAGGAGAAGATAG	61.0	
*MC1R*-F	CGCGGTCACCATCATCG	60.3	98
*MC1R*-R	TGGACTGGCGTCTGCTTTTA	61.4	
*CHP2*-like-F	AATTTGCTTTCCAGCTGTATGAC	60.0	97
*CHP2*-like-R	TGTGATCGATGTCCACTTTCTC	60.7	
*MLPH*-like-1-F	CGCTACAAAGTGATGAGGAAGAG	61.6	97
*MLPH*-like-1-R	CTAACCAGCTCTAGTGGCATAC	60.9	
*MLPH*-like-2-F	CAAGGTCATGAGGTCACTCTAC	60.8	98
*MLPH*-like-2-R	TCCACGTCGTTCCTGTAATG	60.5	
*MITF*-like-F	ATGCTTCGTATACAGGAGTTGG	60.0	97
*MITF*-like-R	AATCTGGCCGAAGGTTATGG	60.9	
*EpoR*-like-F	TGTTCGACTTCTGGCTCATTTC	61.4	96
*EpoR*-like-R	CCACTTTCTCCAGTGACTTCTTG	61.8	
*SOX10*-F	TCGGGGAAAGCAGGTGAT	60.3	100
*SOX10*-R	TGGGGGAAGATATTGGTCAAA	58.3	
*CatS*-F	CAATATTGGGAAATGGACATGGG	60.3	98
*CatS*-R	CATCAATCGCTACTGAAATGGG	60.0	
*MAPK11*-F	GAGAATCATGGAGGTGGTTGGG	64.0	98
*MAPK11*-R	GTGGTACTGTGAGAAATATGGATGGG	63.6	
β*-actin*-F	CACTGTGCCCATCTACGAG	61.1	99
β*-actin*-R	CCATCTCCTGCTCGAAGTC	60.8	

*TYR, tyrosinase; MC1R, melanocortin 1 receptor; CHP2-like, calcineurin B homologous protein 2-like; MLPH-like-1, melanophilin-like-1; MLPH-like-2, melanophilin-like-2; MITF-like, microphthalmia associated transcription factor-like; EpoR-like, erythropoietin receptor-like; SOX10, Sry-related HMg-Box gene 10; CatS, Cathepsin S; MAPK11, mitogen-activated protein kinase 11.*

### Statistical Analysis

Gene expression data were analyzed by one-way analysis of variance (one-way ANOVA) using SPSS 17.0 (SPSS Inc., United States) and GraphPad Prism 8 (GraphPad Software, San Diego, CA, United States). When overall differences were significant, Tukey’s test was conducted to compare the means between individual treatments. Differences were considered significant and highly significant at *p* < 0.05 and *p* < 0.01, respectively.

## Results and Discussion

### Histological Observation and Analysis of Skin

**FIGURE 1:**
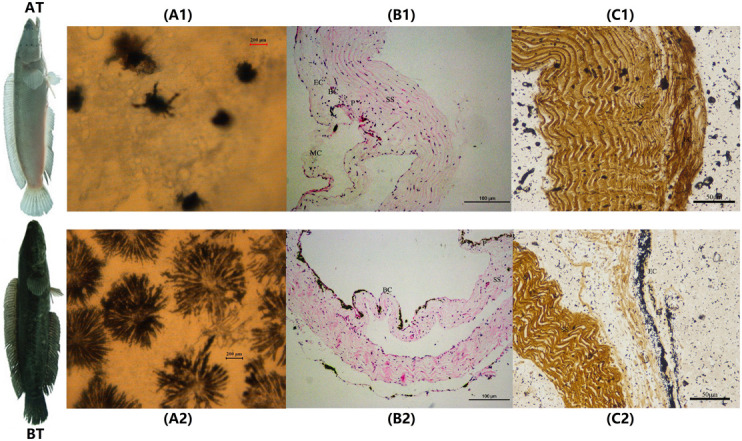
The microscopic and staining analyses of skin melanocytes of two distinct color morphs of *C. argus*. **(A1,A2)** Light microscope observation of skin; **(B1,B2)** H&E staining; **(C1,C2)** Masson Fontana staining. A1, B1 and C1 are albino-type *C. argus* (AT); A2, B2 and C2 are bicolor-type *C. argus* (BT). EC, epithelial cells; MC, mucous cells; BC, basal cells; P, pigmented layer; SS, stratum spongiosum.

The melanocytes in the skin of two distinct color morphs of *C. argus* were observed under an inverted microscope (20×) and results showed that the main differences were reflected in their distribution, density, and size, as well as in the dendritic branching and the growth state of melanocytes. Previously, the body color and markings of fish were determined by the number, the distribution area and the state of pigment particles in pigment cells (Huang, 2008). The shape of the melanocytes in the epidermal layer of *Polyodon spathula* was irregular between the epidermal cells, while they were relatively regular in the dermis (Zarnescu, 2007). Moreover, the melanocytes in the skin of *Xiphophorus meyeri* presented two types, one was with obvious branches and other was contrary (Zheng et al., 2014). In the present study, the melanocytes showed very different morphological characteristics for two color morphs of *C. argus*. In the skin of AT, the density of melanocytes was very small, the distribution was uneven; and the main features were the decrease or disappearance of dendritic branches, these indicated that the development of the melanocyte is hampered and regulated by a series of key albinism-related genes (Yang and Johnson, 2006; Wang et al., 2008; Walderich et al., 2016; Weiner et al., 2019) (Figure 1A1). While in BT, the melanin cells were dense and evenly distributed, the dendritic branches were numerous and relatively thick, the size was large and the color was darker, and many melanocytes were in the growth phase (Figure 1A2). Previously, the H&E and Masson Fontana staining were used to observe the microstructure of the cross section of fish skin, and the distribution and enrichment of melanin (Iaria et al., 2019; Liu et al., 2019; Monteiro et al., 2019). Our results by these two staining techniques demonstrated that the skins of two distinct color morphs of *C. argus* were composed of epidermis, tomb membrane, pigment layer, dermis, subcutaneous tissue, and with obvious boundaries, albeit the epidermisin AT was significantly thin, and the pigment layer tended to degenerate without obvious melanocytes. Conversely, the melanocytes in the pigment layer of BT were relatively concentrated with even and abundant distribution (Figures 1B,C).

### Transcriptome Analysis

The purpose of transcriptomics is to study all the transcripts of specific cells, tissues or organs during a specific development period (Wang et al., 2009). RNA-seq technology can quickly obtain more comprehensive transcriptome expression information in a specific period and growth state based on new generation high-throughput sequencing (Brenner et al., 2000; Lin et al., 2019). The skins of two Oujiang carp with different body color phenotypes were detected by RNA-Seq and 63 SNP sites were obtained for red and white carp (Wang et al., 2014). Moreover, high-quality transcriptome data were obtained from 12 tissues of *Triplophysa rosa* to explore the genetic basis of the albinism by using RNA-Seq technique (Xiao, 2017). The reliable transcriptome information of three phenotypic *Pristella maxillaris* was obtained to analyze molecular regulation of the body color (Bian, 2019). In the present study, the Illumina HiSeq sequencing of two distinct color morphs of *C. argus* generate a total of 56,039,701 (BT) and 60,410,063 clean reads (AT) (*n* = 3) with a mean histogram of insert sizes length (324.91 ± 4.74 bp) and (328.83 ± 4.44 bp), respectively. The percentages of GC Content were (48.12 ± 1.23)% and (48.51 ± 0.71)% and the Phred values greater than or equal to 30 as percentages of the total bases (Q30) were (93.27 ± 0.41)% and (93.01 ± 0.32)%, respectively. After being aligned with the reference genome (GeneBank: SRP078899), the aligned reads were 33,420,418 ± 5,911,223 and 34,739,940 ± 4,998,924, and the comparison efficiencies were (88.99 ± 9.79)% and (86.94 ± 7.27)%, the exonic reads were 13,556,090 ± 2,237,166 and 14,714,228 ± 2,085,045; the intronic reads were 7,123,563 ± 1,233,264 and 7,118,554 ± 1,020,042; the intergenic reads were 9,903,693 ± 1,928,563 and 9,978,757 ± 1,443,047, respectively. The overall statistics of transcript reconstruction results showed that the genes and mRNAs between the reference and query were 20541, 27134 and 20541, 43201; and the proportion of novel exons, novel introns, and novel loci were 12.9, 4.5, and 24.4% based on transcript reconstruction and comparison with the annotation of the previous reference sequence. The transcript reconstruction is conducive to more complete and accurate gene reconstruction, and to better predict gene expression levels (Blanquart et al., 2016; Liu and Dickerson, 2017; Song et al., 2019). The analysis of SNP loci showed that the SNP number, transition, transversion, Ti/Tv, heterozygosity and homozygosity were 46,363 ± 873 and 44,947 ± 392, 27,221 ± 755 and 26,404 ± 245, 19,141 ± 120 and 18,543 ± 162, 1.42 ± 0.03 and 1.42 ± 0.01, 30,107 ± 696 and 27,971 ± 399, and 16,256 ± 178 and 16,976 ± 14. The analysis of alternative splicing shows that the number of all the alternative events is 13396. The summary of transcriptome assembly is shown in Table 2. We found that the quality control effect was relatively high and there was no difference. The clean Data of AT were more than BT, and the mapping data also showed no significant difference. But the quantity of new genes and mRNAs were significantly higher than that of the reference. The analysis of gene structure showed that all the indicators of SNP in BT were higher than that of AT. These results indicate that the BT has a higher genetic diversity during the species evolution (Zhou et al., 2018, 2019). The data of RNA-seq have been submitted to NGDC (https://bigd.big.ac.cn/) under bioproject accession number: PRJCA002700. And the upload information can be found in https://bigd.big.ac.cn/gsa/s/a37YRDtz.

**TABLE 2:** Summary of transcriptome assembly after Illumina HiSeq sequencing of two distinct color morphs *C. argus*.

**Assembly Features**	**BT**	**AT**
**Clean Data**	X ± SD	X ± SD
Read Number	18,679,900 ± 1,366,626	20,136,687 ± 4,002,559
Base Number (bp)	5,603,970,100 ± 409,987,999	6,041,006,300 ± 1,200,767,725
GC Content%	48.12 ± 1.23	48.51 ± 0.71
% ≥ Q30	93.27 ± 0.41	93.01 ± 0.32
**MappingData**		
Aligned reads	33,420,418 ± 5,911,223	34,739,940 ± 4,998,924
Exonic reads	13,556,090 ± 2,237,166	14,714,228 ± 2,085,045
Intronic reads	7,123,563 ± 1,233,264	7,118,554 ± 1,020,042
Intergenic reads	9,903,693 ± 1,928,563	9,978,757 ± 1,443,047
Histogram of insert size (bp)	324.91 ± 4.74	328.83 ± 4.44
Genes (Query/Reference)	27134/20541	
mRNAs (Query/Reference)	43201/20541	
Novel exons	35247/273952 (12.9%)	
Novel introns	10409/230776 (4.5%)	
Novel loci	6624/27134 (24.4%)	
**SNP loci**		
SNP number	46,363 ± 873	44,947 ± 392
Transition	27,221 ± 755	26,404 ± 245
Transverrsion	19,141 ± 120	18,543 ± 162
Ti/Tv	1.42 ± 0.03	1.42 ± 0.01
Heterozygosity	30,107 ± 696	27,971 ± 399
Homozygosity	16,256 ± 178	16,976 ± 14
**Alternative splicing**	Number	
Exon skipping events	5504	
Alternative acceptor sites	3386	
Alternative donor sites	2989	
Intron retention events	1517	
All events	13396	

### Gene Expression Analysis

**FIGURE 2:**
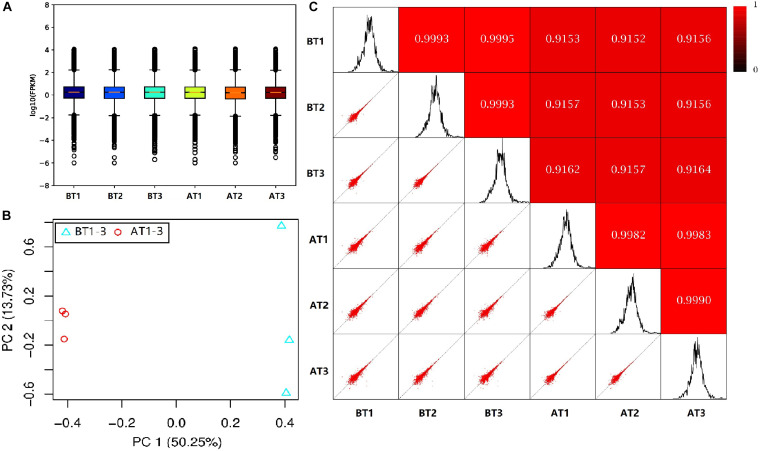
The box plot of the FPKM values **(A)**, PCA cluster diagram **(B)**, and Pearson correlation **(C)** for BT1-3 and AT1-3 *C. argus*. **(B)** PC1 and PC2 represent the first and second principal component; the percentages in parentheses indicate the contribution of the principal components to the differences in the samples. **(C)** The lower left corner is a scatter plot of expression and the upper right corner is the heat map of the correlation between samples. The color and number represent the degree of relevance.

In order to truly reflect the transcript expression level, the numbers of mapped reads and transcript lengths are normalized. The FPKM acted as an indicator of transcript or gene expression (Shahriyari, 2019). A boxplot was made to see not only the dispersion degree of gene expression level distribution in a single sample, but the comparison of overall gene expression levels in different samples (Hänzelmann et al., 2013), we can see that the dispersion degree of the same biological repeats have a certain difference, while a significant difference between two distinct color morphs (Figure 2A). These indicated that there are differences in overall gene expression levels among different samples. The Pearson’s Correlation Coefficient (*r*) analysis is a key step in analyzing the data of RNA-Seq to assess the reliability of biological repeats, which has been widely used in the RNA-Seq field (Schulze et al., 2012). In our study, the PCA cluster diagram and Pearson’s Correlation Coefficient (*r*) were calculated and constructed among six samples base on the FPKM values (Figures 2B,C). The contribution of samples differences are 50.25 and 13.7% of PC1 and PC2, and the difference among AT1-3 is less than that of BT1-3. Meanwhile, the arrangement of *r* values is 0.9152–0.9995, this indicates that the correlation is very high between each sample.

### Analysis of Differentially Expressed Genes

**FIGURE 3:**
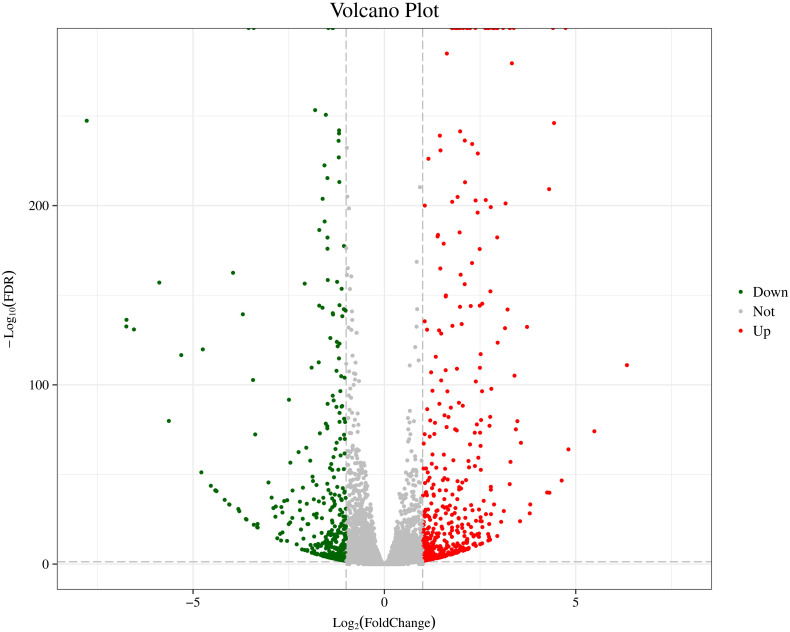
The Volcano Plot of DEGs of two distinct color morphs of *C. argus*. Fold Change: Ratio of expression between the two groups; FDR: Corrected value of the significance difference *p*-value. The gray plots are no DEGs; the green plots are the significantly down-regulated genes, the red plots are the significantly up-regulated genes.

In order to further analyze the DEGs of two distinct color morphs of *C. argus*, we screened the number of DEGs using the AT1-3 as a control group. In previous studies, a large number of DEGs were found in black and light skin of freshwater sticklebacks (Greenwood et al., 2012); and a total of 244 DEGs (177 up- and 67 down-regulated) were found in normal and albino *Triplophysa rosa* (Xiao, 2017); and over 3000 DEGs were obtained in three phenotypic *Pristella maxillaris* (Bian, 2019), and 18,087, 61,751, and 87,737 DEGs between marble and brown trout were grouped by using GO analysis (Djurdjevič et al., 2019). In this study, a total of 1024 DEGs were found, including 559 up- and 465 down-regulated. The Volcano Plot shows that most DEGs are concentrated in ±5-fold change (Figure 3).

**FIGURE 4:**
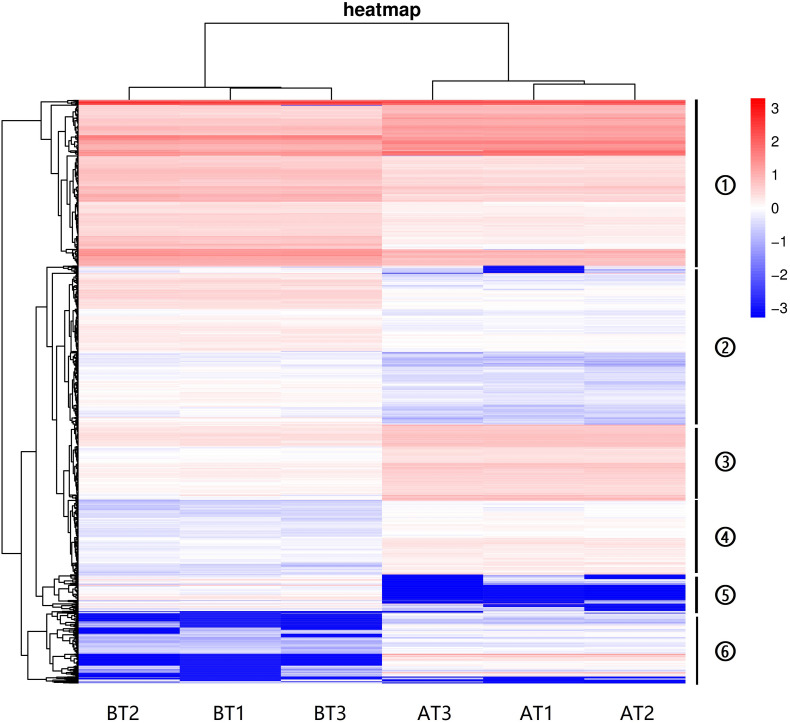
The DEGs heatmap for two distinct color morphs of *C. argus*. Different rows represent different genes. The color represents the level of gene expression in BT1-3 and AT1-3.

The hierarchical cluster analyses have been widely used as tools for exploring gene expression data in RNA-seq, and the results of DEGs heatmap cluster analyses can used to further study the tissue-specific genes and provide insight into gene networks and functions (Severin et al., 2010; Si et al., 2014; Reeb et al., 2015; Kumar et al., 2020). In this study, the DEGs were analyzed by hierarchical clustering of all the samples (Figure 4), and we cluster the genes with the similar expression pattern. The DEGs tree is divided into six parts based on the expression levels [log2 (FPKM + 0.001)]. The parts one and three showed no significant differences, and the parts two, four, five, and six revealed significant differences. These results indicate that the genes show significant expression pattern differences between two distinct color morphs of *C. argus*.

### Functional Annotation and Enrichment Analysis

#### Comparative Analysis of all Genes, New Genes and Differentially Expressed Genes

In order to obtain complete and effective annotation of functional genetic information, different annotation databases were used to analyze the RNA-seq data (Lu et al., 2008; Zheng and Wang, 2008; Ma et al., 2020). Different annotation databases revealed clear differences between the enrichment and annotation information, and GO is the most widely and commonly used among them (Bauer et al., 2008; Eden et al., 2009; Zheng et al., 2019). In the present study, all genes (AGs), new genes (NGs), and DEGs have been annotated functionally, and the number of annotated genes has been counted by using Swiss-Prot, GO, KEGG, COG, KOG, Pfam, and NR databases. We found that the number of AGs, DEGs, and NGs annotated by different databases were extremely different, the NR database exhibited the largest numbers 21820, 767, and 2305, and the COG database has the smallest numbers of 6930, 172, and 184, respectively. Furthermore, the number of different fragment lengths (300 = length < 1000 and length = 1000) showed the same results (Table 3).

**TABLE 3:** Functional annotation and enrichment analysis of all genes of two distinct color morphs of *C. argus* based on different databases.

**Annotation Database**	**Number of genes**			**300 bp ≤ length < 1000 bp**			**Length ≥ 1000 bp**		
	**AGs**	**NGs**	**DEGs**	**AGs**	**NGs**	**DEGs**	**AGs**	**NGs**	**DEGs**
COG	6930	184	148	1736	80	41	5127	84	106
KEGG	9677	392	262	2627	170	85	6848	151	169
GO	9840	517	288	3339	219	127	6221	206	148
KOG	14879	677	359	4121	313	126	10455	252	225
Swiss-Prot	16485	813	548	4616	368	200	11546	332	335
Pfam	18561	848	579	5504	392	212	12685	338	357
NR	21820	2280	765	7148	1067	314	13876	827	422
All	21851	2305	767	7167	1080	316	13885	836	422

*AGs, all genes; NGs, new genes; DEGs, differentially expressed genes.*

**FIGURE 5:**
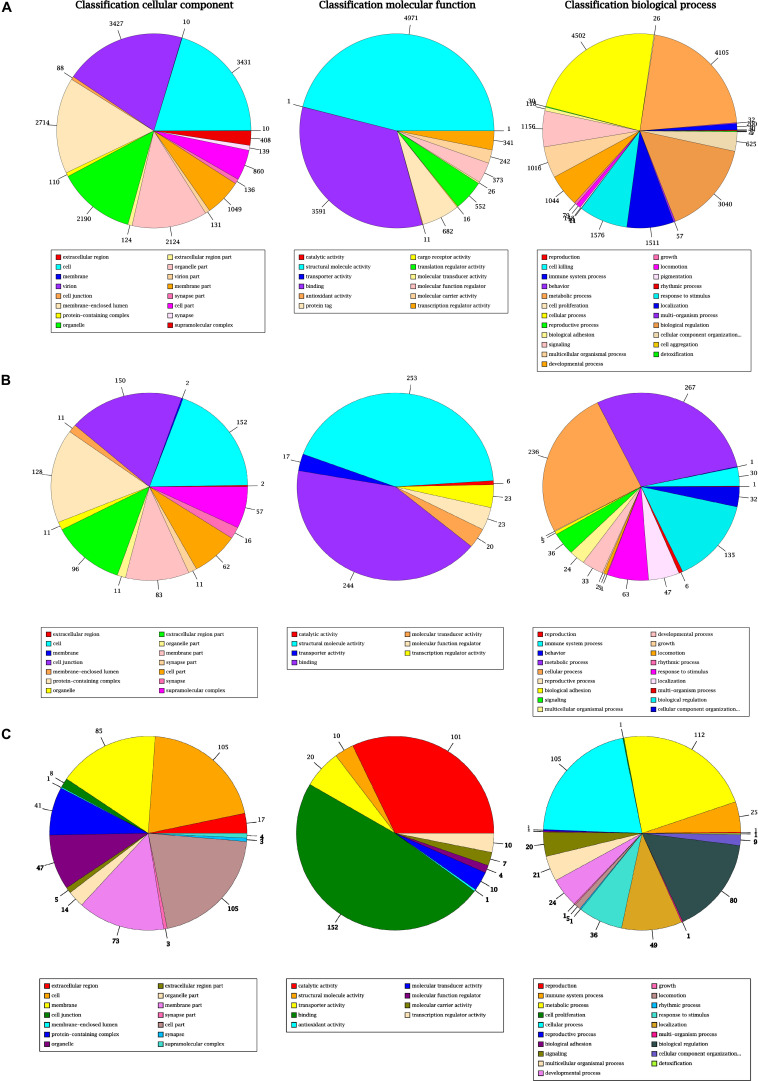
The annotated AGs **(A)**, NGs **(B)**, and DEGs **(C)** were classified into the cellular component, molecular function and biological process according to the GO terms.

GO enrichment analysis of AGs, DEGs, and NGs showed that the cellular component, molecular function and biological process had different gene enrichment and distribution trends (Young et al., 2010; Kim et al., 2019). In the classification of cellular component, the largest proportion of secondary functions genes were enriched in cell (3431), virion (3427), membrane-enclosed lumen (2714), organelle (2109), organelle part (2124), and membrane part (1049); for AGs (≥1000), in cell (152), cell junction (150), protein-containing complex (128), extracellular region part (96), membrane part (83); for NGs (≥80), and in cell (105), membrane (85), membrane part (73), and cell part (105); for DEGs (≥70), which indicate that the number of NGs and DEGs were obviously consistent with that of AGs. In the classification of molecular function, the largest proportion of secondary functions genes were enriched in structural molecule activity (4971 and 253), binding (3591 and 244) both for AGs (≥ 1000) and NGs (≥80), but in catalytic activity (101) and binding (152) for DEGs (≥70). In the classification of biological process, the largest proportion of secondary functions genes were enriched in cellular process (4502), metabolic process (4105), biological regulation (3040), response to stimulus (1576), locallzation (1511), signaling (1156), multicellular organismal process (1016), developmental process (1044); for AGs (≥1000), and in metabolic process (267 and 112), cellular process (236 and 105), biological regulation (135 and 80) both for NGs (≥80) and DEGs (≥70), respectively. These results reveal that all the secondary functions of genes in NGs and DEGs are belong to AGs, and the metabolic and cellular process, biological regulation are the most important factor between two distinct color morphs of *C. argus*. The GO annotation classification statistics are showed in Figure 5.

#### GO Analysis of DEGs

**FIGURE 6:**
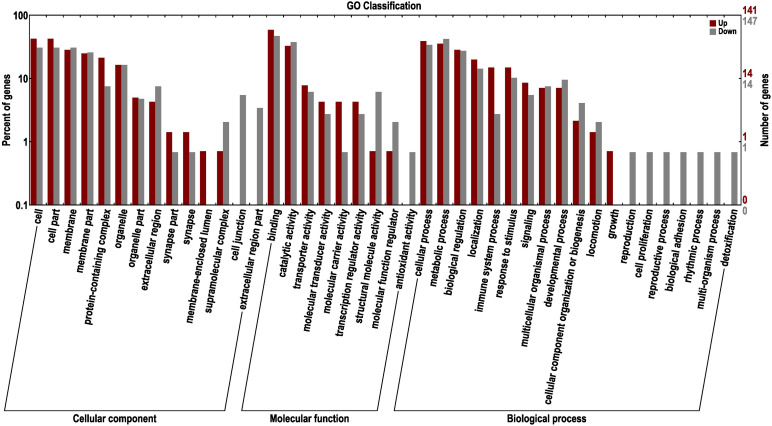
GO classification of up-regulated and down-regulated genes in the BT group, compared to the AT group. The left panel shows the proportions of up- and down-regulated genes according to the GO terms. The right panel shows the numbers of up- and down-regulated genes.

To further excavate the functional genes with significant differences, the up- and down-regulated genes were selected for enrichment and annotation based on AT1-3 vs BT1-3 (Figure 6). We can see that there are 465 down-regulated and 559 up-regulated genes in the BT group, compared to the AT group. In other studies focusing on skin color showed that the DEGs of three different skin colored Red Tilapia showed significant difference (Zhu et al., 2016), there are 3683 and 3434 genes that were up-regulated in red and white crucian carp based on FDR < 0.0001 and |log2 (Fold Change)| ≥ 1 (Zhang et al., 2017), and a total of 785 unique genes (385 up-regulated and 400 down-regulated genes) were differentially expressed in albino individuals of *Acipenseriformes gueldenstaedtii* (Gong et al., 2019). Meanwhile, the topGO analysis (Alexa and Rahnenfuhrer, 2010) show the most significant enrichment of 10 nodes are peptide antigen binding, oxygen binding, oxygen transporter activity, threonine-type endopeptidase activity, serine-type endopeptidase activity, guanylate cyclase activity, miRNA binding, RasGTPase binding, lipid binding and carboxy-lyase activity.

**FIGURE 7:**
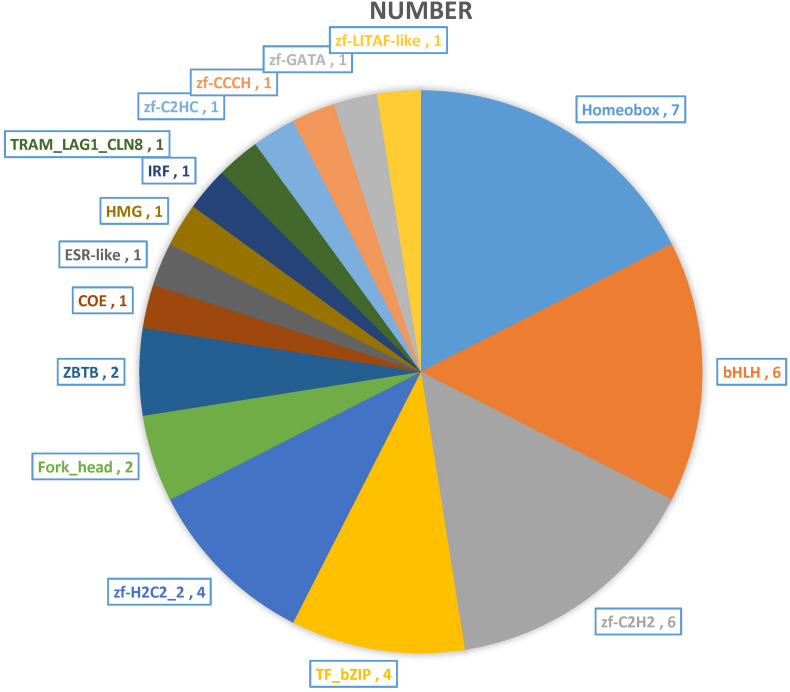
The transcription factors (TFs) of DEGs between two distinct color morphs of *C. argus*.

The transcription factors (TFs) regulate many cellular processes, which can repress or activate the transcription of target genes (Berest et al., 2019). The activity of signaling pathways can be replaced by the change of TFs activity (Kim et al., 2007; Whyte et al., 2013). And the TFs can act as transcriptional activators and repressors (Han et al., 2018). The TFs of DEGs are also annotated and mined in this study. A total of 40 TFs were found and classified to 16 TFs families (Figure 7). The Homeobox (7), bHLH (6), zf-C2H2 (6), TF_bZIP (4), and zf-H2C2_2 (4) have large numbers of distributions. Previous studies have shown that the basic helix-loop-helix-leucine zipper (bHLH-ZIP) can activate tyrosinase (TYR) promoter E-box, and then activates the expression of TYR gene and its related proteins, and TYR is the most critical enzyme in melanin synthesis (Hodgkinson et al., 1993; Ferguson and Kidson, 1996; Steingrímsson et al., 2004). Our results in accordance with previous studies provide good molecular clues for the albino body color of AT. At the same time, the TFs that regulate immunity were also found, such as interferon regulatory factor (IRF), which can positively regulate IFN expression and play an extremely important role in the fish immune system (Honda and Taniguchi, 2006; Tamura et al., 2008; Zhao et al., 2020).

#### COG and KOG Analysis of DEGs

**FIGURE 8:**
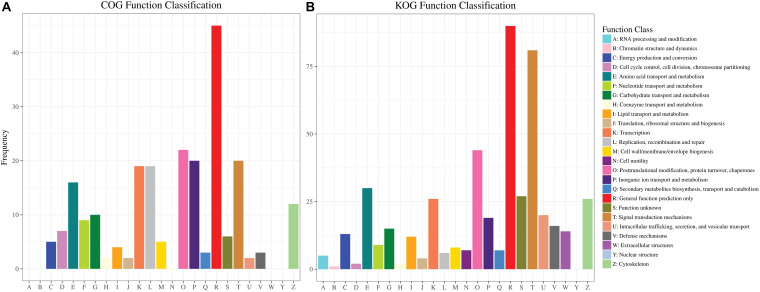
Clusters of COG and KOG of DEGs in two distinct color morphs of *C. argus.* **(A,B)** COG and KOG function classification of DEGs.

Cluster of Orthologous Groups and KOG are databases for prokaryotic and eukaryotic species, which are also employed for orthologous classification of protein sequences (Luan et al., 2017; Nie et al., 2020). Cluster of Orthologous Groups and KOG analysis could provide useful information about the possible function of DEGs (Figure 8). A total of 184 (COG) and KOG (419) with 25 functional definitions were obtained.

#### KEGG Analysis of DEGs

**FIGURE 9:**
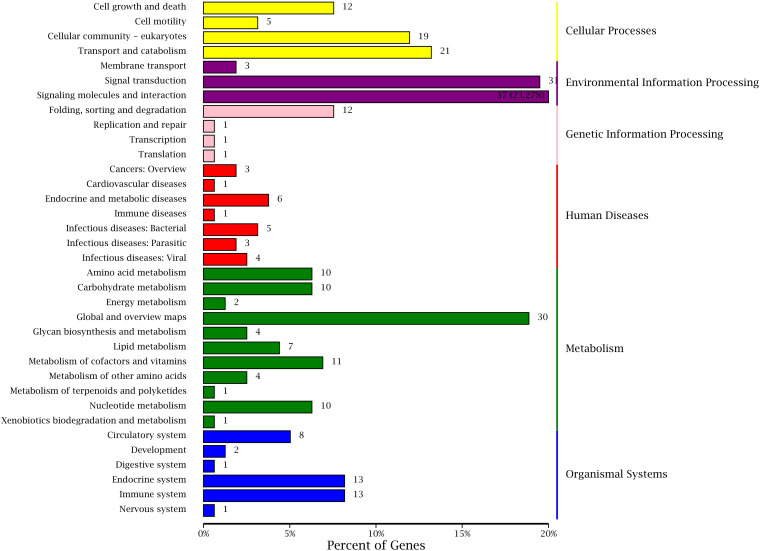
KEGG classifications of DEGs. The right and left ordinate is the primary and secondary metabolic pathway of KEGG; the abscissa is the number of genes annotated to the KEEG pathway and their proportion.

Kyoto Encyclopedia of Genes and Genomes is a genomic information database with a systematic analysis of gene function, and it can identify the most important biochemical metabolic pathways and signal transduction pathways involved in DEGs (Kanehisa and Goto, 2000; Aoki and Kanehisa, 2005). The annotation results of DEGs were classified according to the pathway types in KEGG of BT vs AT group (Figure 9). A total of 262 DEGs were assigned to 130 different pathways in present study, and six primary and 35 secondary metabolic pathways were classified. Meanwhile, the signal transduction (31) and signaling molecules and interaction (37) of environmental information processing, global and overview maps (30) of metabolism showed the highest proportion according to the KEGG pathway classification. These results indicate that the environmental factors may have a huge contribution to the formation of different body colors of aquatic species (Cheng, 2008; Yadufashije and Samuel, 2019), and we inferred that the different body colors of *C. argus* might be closely related to the geographical environment, but further research and verification need to be investigated.

**FIGURE 10:**
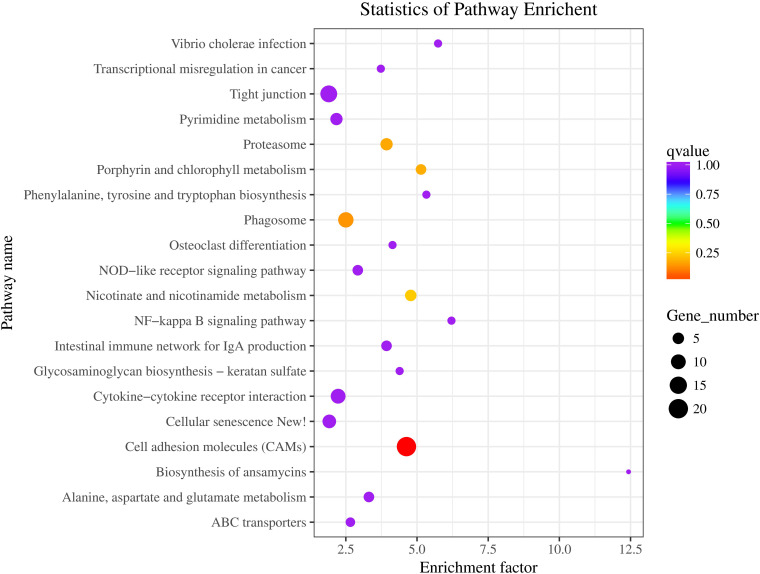
The statistics of top 20 most reliable enriched pathways. Pathway Name: KEGG tertiary metabolic pathway; Q-value: Significance statistics of enrichment, the smaller the Q-value, the higher the degree of enrichment.

The statistics of pathway enrichment are measured by using enrichment factor, Q-value and the number of genes enriched in the pathway. The top 20 most reliable enriched pathways were screened and identified according to the DEGs of BT vs AT group (Figure 10). We can see that the most numbers and the highest degree of enrichment genes were associated with cell adhesion molecules pathway, and then with the phagosome pathway, and the phenylalanine, tyrosine and tryptophan biosynthesis pathway, this provides clues for the mechanism of skin albinism and also points out the direction for subsequent research. The DEGs of two different carps were mainly enriched in the melanin synthesis, WNT and MAPK signaling pathways by using transcriptome detection and these DEGs may participate in the formation of body color (Jiang et al., 2014). 46 DEGs related to body color were screened out from the skins of different body color (Henning et al., 2013). Meanwhile, some immune-related pathways, such asNF-kappa B signaling pathway, intestinal immune network for IgA production pathway, and vibrio cholerae infection pathway, may be closely related with the collected tissue of skin. In a previous study, we found that the skin of *Channa* could secrete a large amount of mucus under stress to stimulate its non-specific immune function (Zou and Zhuo, 2010). These results also indicate that there is difference in immunity between two distinct color morphs of *C. argus*.

### Validation of the Data Reliability by qRT-PCR

**FIGURE 11:**
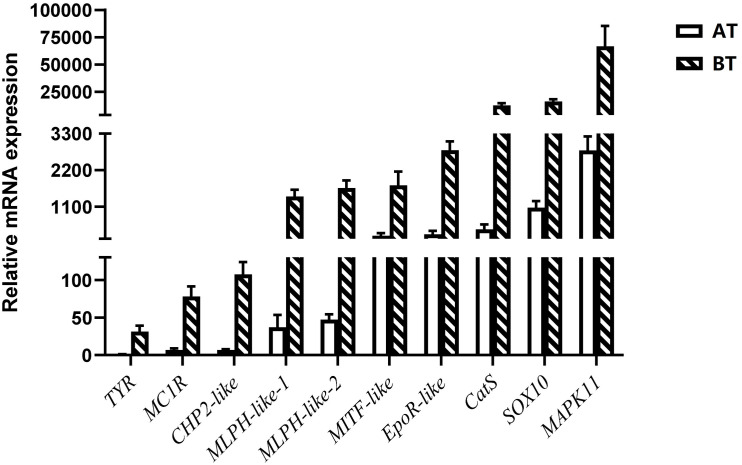
Relative mRNA expressions of DEGs according to RNA-seq focus on pigment-related genes.

**FIGURE 12:**
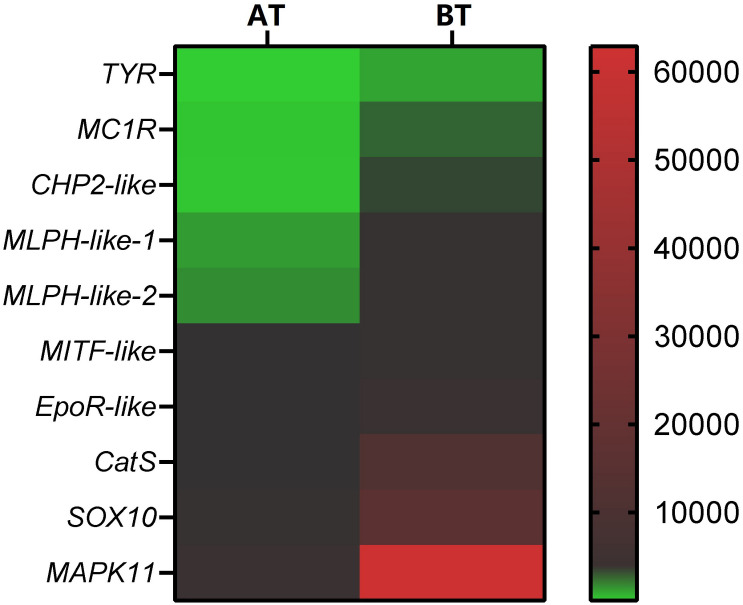
Heatmap of DEGs according to RNA-seq focus on pigment- related genes. The smallest value of green is 0.93, the baseline value of black is 120, and the largest value of red is 62881.

In order to verify the RNA-seq results, ten pigment-related genes (Casp et al., 2002; Bhoumik et al., 2007; Mirmohammadsadegh et al., 2010; Liu et al., 2013; Hammam et al., 2014; Wang et al., 2017; Yang et al., 2018; Li et al., 2020) were selected to quantify their mRNA expression levels in the skin of two distinct color morphs of *C. argus* by using qRT-PCR (Figure 11). The expressions of all the selected genes of BT were extremely higher than that of AT, consistent with the results obtained through transcriptome analysis. Moreover, the development of melanocytes is controlled by many factors and these factors cooperate with each other to form a gene network that regulates the development and differentiation of melanocytes derived from neural crests (Setty et al., 2007; Mort et al., 2015). The TYR is a key and rate-limiting enzyme that controls melanin synthesis, the low expression level of TYR may directly and closely relate to the albinism of the skin (Koga and Hori, 1997; Li et al., 2014; Hirobe and Ishikawa, 2015; Kaur and Dua, 2015; Jiang et al., 2019). Meanwhile, the MITF is an upstream TF that can directly regulate the TYR gene (Bharti et al., 2012; Raviv et al., 2014), and some genes such as SOX10, MAPK, CATs act directly or indirectly on the MITF gene to regulate the development, growth and differentiation melanocytes (Hou et al., 2000). In addition, the MLPH, CHP2, and EpoR play roles in the formation, transportation, development and other processes of pigment cells and melanoma (Bhoumik et al., 2007; Liu et al., 2013; Hammam et al., 2014; Li et al., 2020). Previous studies showed that the differential expression of albino-related genes regulated the development and differentiation of body color, and over 95 and 38 body color development related genes were found in zebrafish and *Oryziaslatipes* (Hidehito et al., 1994; Dooley et al., 2013). The development, differentiation and migration of pigment cells were regulated by multiple genes and complex gene regulatory networks (Kelsh et al., 2000; Ceinos et al., 2015). As a key enzyme for controlling the melanin synthesis, the expression and activity of *TYR* directly determine the rate and yield of melanin production (Ito et al., 2000). *MITF* and *SOX10* are the core transport regulators in pigment cell development, the *MITF* activates TYR gene expression by acting on the TYR promoter E-box, and *SOX10* acts as a powerful activator to directly regulate the expression of *MITF* (Lang et al., 2005; Sarkar and Hochedlinger, 2013). Meanwhile, the MLPH-1 and -2 also act as linker proteins between the melanosome and MYO5A-bound actin filament (Provance et al., 2002; Kuroda et al., 2003; Li et al., 2020). Melanocortin 1 receptor (MC1R) plays a key role in the differentiation of adult melanocytes (Ozdeslik et al., 2019). Other albino-related genes also play important roles impacticting on the survival, proliferation, migration and differentiation of the pigment cell (Aizawa et al., 2005; Johnstone et al., 2005; Hammam et al., 2014; Hadrian et al., 2019). The heatmap indicates that there is a significant difference between two distinct color morphs of *C. argus* and the expressions of different genes (Figure 12).

## Conclusion

The regulation mechanism of fish body color is relatively complex, and the influencing factors are also diverse. Genetics, habitat, neuroendocrine and feed nutrition are considered to be the most important factors. Observation of skin histology can intuitively show the albino characteristics of different color types of *C. argus*, and serves to help understand the internal mechanism of albinism. Our results imply that the lack of melanocytes and melanin deposits, the low density and imperfect development of melanosome in skin are the histological reasons that cause the albinism of *C. argus*. As an effective and reliable molecular biology technique, the Illumina HiSeq sequencing can discover key functional genes and their regulatory networks. And a total of 767 DEGs were screened from the transcriptome data, most of them were distributed in the functions of cell (210), catalytic activity (101) and binding (152), metabolic and cellular process (112 and 105), biological regulation (80), including a large number of DEGs involved in body color formation, which were located in the melanin synthesis, WNT and MAPK signaling pathways. Meanwhile, the expression of ten key pigment-related genes in skins of two color type of *C. argus* were compared and verified, the results showed that their low expression was one of the key factors for the appearance of whitening characteristics. These findings indicate that the RNA-Seq technology can be well used to study the genetic mechanism of fish body color formation. In addition, our results can provide a molecular basis to further reveal the regulation mechanism of fish body color development and theoretical support for the protection and development of rare germplasm resources.

## Data Availability Statement

The datasets generated for this study can be found in NGDC (https://bigd.big.ac.cn/) under bioproject accession number: PRJCA002700.

## Ethics Statement

The animal study was reviewed and approved by the Animal Care Committee of South China Agricultural University (Guangzhou, China).

## Author Contributions

JZ and AZ contributed to the study design, analysis and interpretation of data, and drafting/revising the manuscript. AZ and DS performed the sample collection. SX, YF, and SL performed the histology experiment. DS and ZS performed the gene expression experiment. AZ, ML, and CZ contributed to the analysis of the data and wrote the manuscript. All authors read and approved the final manuscript.

## Conflict of Interest

ML was an independent researcher during our study and was subsequently employed by Genepioneer Biotechnologies.

The remaining authors declare that the research was conducted in the absence of any commercial or financial relationships that could be construed as a potential conflict of interest.

## Footnotes

1 http://genome.crg.es/astalavista/
